# Syphilitic Iritis

**Published:** 1891-07

**Authors:** Charles Dunbar Roy

**Affiliations:** Atlanta, Ga.


### SYPHILITIC IRITIS.

By CHARLES DUNBAR ROY, A. B., M. D., ATLANTA, GA.

Syphilis as a cause of iritis is very common. Noyes states that syphilis is the cause of thirty per cent. of all cases. Mooren, of Wiesbaden, states that out of 2,068 cases of iritis only 169 were purely syphilitic. This latter percentage is, I think, rather small in comparison to that of America. Statistics taken by me while house surgeon of the ophthalmic wards of Charity Hospital, New York, showed a percentage of 25. Of course the character of the cases must be taken into consideration, since they were those of the lowest order and who had possessed the worst hygienic surroundings. Ratio of frequency of the disease between men and women was found to be 1.75 to 1, respectively. All cases occurred with syphilis in the secondary stage, the third stage being much the less common. Widder, in Graefe's Archives Ophth. vol. xxvii., p. 99, says that specific affections are local manifestations of the constitutional syphilis; iritis is very common in the second stage, occurs in the early phase of syphilis and is a product of the coadylomatous stage. Gummous iritis occurs in nineteen per cent. of all cases of iritis. The severer the syphilitic eruption is, the severer will be the eye symptoms.

In the pustular syphilide the iritis was always severe. Another distinctive feature of specific iritis is its decided plastic form. The products of its inflammation are very tenacious, and the agglutination of the iris to the lens capsule is very prone to occur. In every case this tendency to adhesion is one point which must always be borne in mind in its treatment. The frequency of syphilis as a cause of iritis compels the physician to inquire minutely into the history of every case which comes under his care. If the patient should have a rheumatic or malarial dyscrasia, the cases may prove much more tedious in their treatment. Especially is photophobia prone to persist in the weak and anæmic, for while the reaction of the pupil is normal, the general past hyperæmic state of the uveal tract causes the retina to be much more sensitive to light impressions. The objective and subjective symptoms of specific iritis will not furnish a perfect criterion by which to diagnose its special form, for they differ in no respect from other forms. If there is any one point which is especially significant of this form of iritis it is the excessive circumcorneal injection and the tendency for the exudation from the engorged vessels to raise the conjunctiva around the cornea into bleb-like elevations. The treatment of specific iritis must be prompt and energetic. Combined local and internal remedies are always necessary. Atropine must be pushed until some impression is made upon the iris. A solution of either 2 grains or 4 grains to the ounce may be used. Begin by instilling two drops of the solution into the affected eye every three hours, and if no mpression is made upon the iris in twenty-four hours increase the frequency to every hour, and even to every half hour, watching, however, for any toxic symptoms. As an adjuvant to the above there is nothing equal to hot water applied by dipping sheet lint into water as hot as can be borne, and allowing it to lie upon the eye until cool, to be again renewed. This is done for twenty minutes at a time, every two or three hours, depending upon the intensity of the pain. There is no anodyne equal to the above, and in relieving the congestion it is often more efficacious than the leeches. If the latter method does not succeed in ameliorating the symptoms, and great congestion still exists, two to three leeches are applied to the temple of the affected eye. In using this latter procedure the æsthetic tastes of the patient should not be disregarded. The internal treatment must be pushed to its full effect. Mercury in some form is always used, especially as this is nearly always the manifestation of the secondary stage. Inunctions may be used if the stomach is sensitive, but I prefer the following pill:

℞. Hydrarg. Protoid., gr. v.
Ext. Hyoscyami, gr. vi.
Ferri et Quininæ cit., ʒi.
M. Ft. capsules No. xxx.

Sig. One three times daily; increase until five are taken daily. This latter will be found very effective. The hypodermic method of administration now has many advocates but experience with it has not been sufficient to allow me to speak with authority. A daily observation of the patient by the physician is of great assistance to both, for though the directions may have been explicit, one often finds the omission of some important *sine qua non.*

14½ *Whitehall St.*

